# Age-stratified signals and early onset of adverse events with pregabalin: a disproportionality analysis of the FDA adverse event reporting system

**DOI:** 10.3389/fmed.2026.1806099

**Published:** 2026-10-01

**Authors:** Jianghai He, Yuwen Hu, Hesong Pan, Ziyang Jin, Zheng Tu, Hongyu Ye, Caixiang Zhuang, Qingxiang Yu, Xiangting Wang, Yanyan Zheng, Qiong Yao

**Affiliations:** 1 Department of Neurology, Postgraduate Training base Alliance of Wenzhou Medical University (Wenzhou People’s Hospital), Wenzhou, Zhejiang, China; 2 Wenzhou Third Clinical Institute Affiliated to Wenzhou Medical University, Wenzhou People’s Hospital, Wenzhou, Zhejiang, China; 3 The Affiliated Changsha Hospital of Xiangya School of Medicine, Central South University, Changsha, Hunan, China

**Keywords:** adverse event, disproportionality analysis, FAERS database, pharmacovigilance, pregabalin

## Abstract

**Introduction:**

Pregabalin is widely used in the management of neuropathic pain and generalized anxiety disorder. However, comprehensive real-world evidence regarding its neuropsychiatric safety profile and age-specific risk variations remains insufficient.

**Methods:**

Data were retrieved from the FDA Adverse Event Reporting System (FAERS) from Q1 2005 to Q1 2025. Four disproportionality algorithms—reporting odds ratio (ROR), proportional reporting ratio (PRR), Bayesian confidence propagation neural network (BCPNN), and empirical Bayesian geometric mean (EBGM)—were utilized to identify safety signals. The Weibull distribution model characterized the time-to-onset (TTO) of adverse events (AEs) and compared risks across age subgroups.

**Results:**

After analyzing 98,196 pregabalin-related reports, 279 preferred terms generated consistent signals using all four disproportionality methods. Confirmed AEs included dizziness, weight increased, and somnolence. Unexpected signals were identified, such as hypoacusis, dysgraphia, logorrhoea, and feeling of despair. Age-stratified analysis revealed that older adults (≥65 years) showed higher risks for falls, and balance disorder. In contrast, adults (18–64.9 years) more frequently reported withdrawal syndrome, drug dependence, and suicidal ideation. The TTO analysis revealed that 54.53% of AEs emerged during the initial month of treatment. The median onset was shorter in older adults (16 days) than in adults (30 days). The Weibull shape parameters (*β*) for both groups were below 1 (0.46 and 0.48, respectively), indicating an early failure type.

**Conclusion:**

This study identifies novel safety signals and distinct age-related risk patterns. Vigilant monitoring during the initial treatment phase is essential, particularly for neuropsychiatric stability and physical safety in vulnerable populations.

## 1 Introduction

Pregabalin is a widely utilized pharmacological agent. Although initially developed for neuropathic pain and epilepsy (1, 2), it is now frequently prescribed for generalized anxiety disorder (GAD) due to its rapid onset and its role in modulating excitatory neurotransmitter release. Its clinical importance is underscored by the high comorbidity between chronic pain and affective disorders. research indicates that approximately 60% of patients with neuropathic pain experience secondary depressive or anxiety symptoms (3). Consequently, pregabalin has become a common interdisciplinary treatment in both psychiatry and pain management. Mechanistically, pregabalin achieves its therapeutic effect through interaction with the *α*2*δ* subunit of voltage-gated calcium channels, thereby inhibiting central sensitization and alleviating anxiety symptoms (4). While common adverse events (AEs), such as sedation, dizziness, peripheral edema, and weight gain, are well-documented, they represent only a subset of the drug's clinical safety profile (5, 6). Recently, regulatory agencies have issued increasingly stringent warnings regarding the risks of dependence and withdrawal symptoms associated with pregabalin, as well as its potential links to paradoxical mood instability and suicidal ideation (5).

Current global research on pregabalin safety has identified various risks, yet the evidence remains fragmented. Previous studies have primarily focused on isolated AEs. For instance, analyses of the US FAERS database have highlighted risks of abuse, dependence, and overdose (7, 8), while other reports emphasized specific systemic risks such as severe cutaneous adverse reactions (SCARs) in Asian populations (9) or rhabdomyolysis (10). Although some pharmacovigilance efforts have addressed neuropsychiatric signals—including suicidal behavior and psychotic disorders (11, 12)—they often lacked multi-algorithm validation or failed to account for the temporal characteristics (Time-to-Onset) and age-stratified variations of these risks. Consequently, a comprehensive and robust evaluation of pregabalin's overall safety profile, particularly regarding its neuropsychiatric impact in real-world psychiatric settings, is still lacking.

This study utilized the U.S. FDA Adverse Event Reporting System (FAERS) database to conduct a comprehensive evaluation of pregabalin's safety profile using multiple disproportionality algorithms. By mapping the full spectrum of adverse signals, we aimed to specifically clarify the neuropsychiatric risk landscape and identify previously underreported safety concerns that may complicate the management of affective disorders. Furthermore, we employed the Weibull distribution model to characterize the temporal onset patterns of these AEs and explored risk variations across age subgroups. These findings are intended to provide a scientific basis for evidence-based risk mitigation and to promote the rational and safe application of pregabalin in patients with emotional and psychiatric vulnerabilities.

## 2 Materials and methods

### 2.1 Data source and mining

This research followed the READUS-PV guidelines and employed multiple disproportionality analysis methods to perform a thorough post-marketing evaluation of pregabalin's safety profile (13). Data sourced from the quarterly extracts of the FAERS database, can be accessed at the following URL: https://fis.fda.gov/extensions/FPD-QDE-FAERS/FPD-QDE-FAERS.html.

The FAERS database contains key components such as demographic (DEMO), drug (DRUG), adverse event (REAC), outcome (OUTC), source (RPSR), therapy date (THER), and indication (INDI) data. All reports were classified using the Medical Dictionary for Regulatory Activities (MedDRA), version 28.0. This study utilized FAERS data from the first quarter of 2005 through the first quarter of 2025 (Q1 2005–Q1 2025, inclusive). Reports of pregabalin-associated AEs were identified by querying the DRUG file for records designating pregabalin (generic name) or Lyrica (brand name) as the primary suspected (PS) drug. Data cleaning followed U.S. FDA-recommended procedures, Duplicate case reports were removed by retaining the most recent entry based on CASEID and FDA_DT; PRIMARYID was used to resolve ties. To reduce potential competing drug attribution and attribution ambiguity, reports were excluded when selected CNS-active medications, including gabapentin, tramadol, oxycodone, hydrocodone, duloxetine, amitriptyline, trazodone, and alprazolam, or their common trade names (e.g., Neurontin, Ultram, and OxyContin), were recorded as either secondary suspect (SS) or concomitant (C) drugs. These medications were selected because of their overlapping clinical use with pregabalin or their potential to produce neurological or psychiatric adverse-event profiles that could complicate attribution of reported events to pregabalin. This restrictive criterion was intended to obtain a more pregabalin-focused reporting dataset rather than to eliminate residual confounding. Additionally, to reduce heterogeneity arising from clearly non-routine or non-therapeutic use scenarios, reports associated with intentional misuse, intentional overdose, medication errors, off-label use, or related product-use issues were excluded from the primary analysis.

AEs were coded using the Medical Dictionary for Regulatory Activities (MedDRA), version 28.0. Each AE was first coded at the preferred term (PT) level and then mapped to its corresponding system organ class (SOC) according to the MedDRA hierarchy. The 27 SOCs reported in the Results section were derived from this SOC-level mapping of pregabalin-related PTs. Each individual case safety report (ICSR) could contain multiple PTs, all of which were incorporated into the subsequent disproportionality assessments. Available clinical characteristics—such as gender, age, reporting region, outcomes, and reporter type—were extracted. It should be noted that a single case could involve multiple serious outcomes (e.g., death, hospitalization, disability), which means the overall count of severe outcomes could surpass the total number of reported cases. The overall data processing workflow is illustrated in Figure 1.

**Figure 1:**
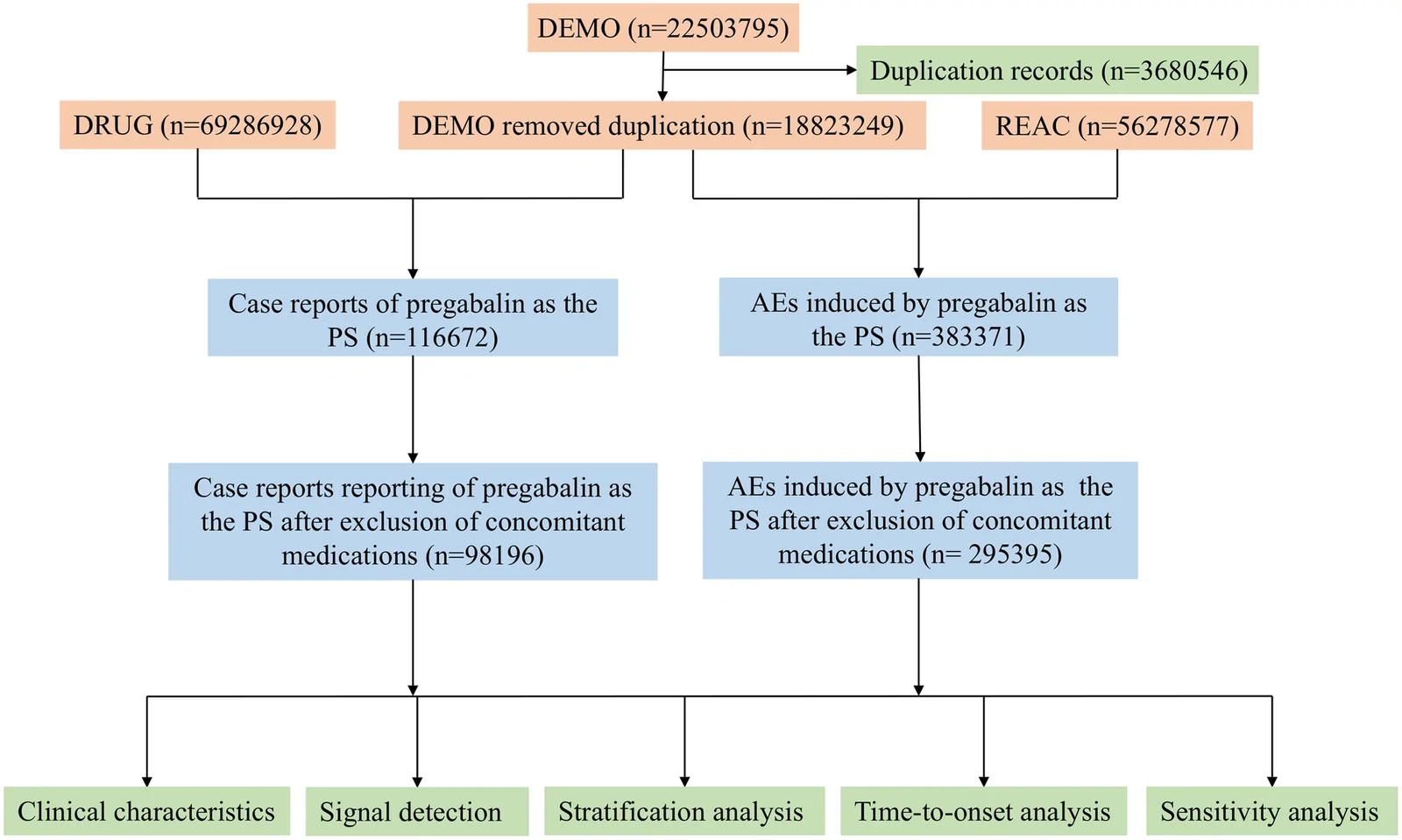
Flowchart for the identification of adverse events associated with pregabalin in the FAERS database. FAERS, United States Food and Drug Administration Adverse Event Reporting System; AEs, adverse events; DEMO, demographic and administrative information file; DRUG, drug information file; REAC, adverse event file; PS, primary suspect.

### 2.2 Statistical analysis

Disproportionality analysis was performed to identify disproportionate drug–adverse event reporting patterns rather than to establish causal relationships. For each PT, a 2 × 2 contingency table was constructed by comparing reports involving pregabalin with all other reports in the FAERS database. The structure and cell definitions of the 2 × 2 contingency table are provided in Supplementary Table 1. Four complementary signal-detection approaches were applied, including reporting odds ratio (ROR), proportional reporting ratio (PRR), Bayesian confidence propagation neural network (BCPNN), and empirical Bayesian geometric mean (EBGM). ROR and PRR are straightforward measures suitable for initial signal screening (14), whereas BCPNN and EBGM provide shrinkage estimates that are generally more stable in the presence of sparse data and may reduce spurious signals arising from small report counts (15, 16). A signal was considered significant only when all four approaches simultaneously met their predefined criteria: at least three reports and a lower bound of the 95% confidence interval for the ROR greater than 1; at least three reports, a PRR of at least 2, and a chi-square value of at least 4; an IC025 greater than 0; and an EBGM05 greater than 2. Requiring concordance across all four approaches was intended to improve the robustness and specificity of signal detection and to reduce the likelihood of false-positive findings. The formulas and detailed computational procedures are provided in Table 1. For the age-subgroup comparison, pregabalin-related reports were divided into adults aged 18–64.9 years and older adults aged ≥65 years. For each selected PT, a 2 × 2 contingency table was constructed within pregabalin-related reports to compare the reporting frequency of that PT between the two age groups. Odds ratios (ORs) with 95% confidence intervals (CIs) were calculated to quantify differences in reporting odds between older adults and adults. An OR > 1 indicated higher reporting odds in older adults, whereas an OR < 1 indicated relatively higher reporting frequency in adults aged 18–64.9 years. Statistical significance was assessed using Pearson's chi-square test or Fisher's exact test, as appropriate. Data mining and statistical evaluations were mainly performed with R software (version 4.4.1).

**Table 1:** ROR, PRR, BCPNN, and EBGM methods, formulas, and thresholds.

Method	Formula	Threshold
ROR	$\;\;\mathrm{ROR}=\frac{a/c}{b/d}$	*a* ≥ 3 and the lower limit of the 95% CI > 1.
	$\mathrm{SE}(\mathrm{lnROR})=\sqrt{\frac{1}{a}+\frac{1}{b}+\frac{1}{c}+\frac{1}{d}}$	
	$95\text{\%}\mathrm{CI}=e^{\ln(\mathrm{ROR})\pm 1.96\mathrm{se}}$	
PRR	$\mathrm{PRR}=\frac{a/(a+b)}{c/c+d}$	*a* ≥ 3, PRR ≥ 2, and χ² ≥ 4.
	$\mathrm{SE}(\mathrm{lnPRR})=\frac{1}{a}-\frac{1}{a+b}+\frac{1}{c}-\frac{1}{c+d}$	
	$95\text{\%}\mathrm{CI}=e^{\ln(\mathrm{PRR})\pm 1.96\mathrm{se}}$	
BCPNN	$\mathrm{IC}=\mathrm{lo}g_{2}\frac{p(x,y)}{p(x)p(\gamma )}=\mathrm{lo}g_{2}\frac{a(a+b+c+d)}{\left(a+b)(a+c\right)}$	IC025 > 0
	$E(\mathrm{IC})=\mathrm{lo}g_{2}\frac{\left(a+\gamma^{11})(a+b+c+d+a)(a+b+c+d+\beta \right)}{\left(a+b+c+d+\gamma )(a+b+\alpha 1)(a+c+\beta 1\right)}$	
	$V(\mathrm{IC})=\frac{1}{{\left(\ln\;2\right)}^{2}}[\frac{(a+b+c+d)-a+\gamma -\gamma^{11}}{\left(a+\gamma^{11}\right)}+\frac{(a+b+c+d)-(a+b)-\alpha 1}{\left(a+b+\alpha 1)(a+b+c+d+\alpha \right)}+\frac{(a+b+c+d+a)-(a+c)+\beta -\beta 1}{\left(a+b+\beta 1)(1+a+b+c+d+\beta \right)}$	
	$\gamma =\gamma^{11}\frac{\left(a+b+c+d+a)(a+b+c+d+\beta \right)}{\left(a+b+\alpha 1)(a+c+\beta 1\right)}$	
	$\mathrm{IC}-2\mathrm{SD}=E(\mathrm{IC})-2\sqrt{V(\mathrm{IC})}$	
EBGM	$\mathrm{EBGM}=\frac{a(a+b+c+d)}{\left(a+b)(a+c\right)}$	EBGM05 > 2
	$\mathrm{SE}(\ln\;\mathrm{EBGM})=\sqrt{\frac{1}{a}+\frac{1}{b}+\frac{1}{c}+\frac{1}{d}}$	
	$95\text{\%}\mathrm{CI}=e^{\ln\;(\mathrm{EBGM})\pm 1.96\mathrm{se}}$	

N, the number of reports; ROR, reporting odds ratio; PRR, proportional reporting ratio; 95% CI, 95% confidence interval; *χ*^2^, chi-squared; IC, information component; IC025, the lower limit of 95% CI of the IC; EBGM, empirical Bayesian geometric mean; EBGM05, the lower limit of 95% CI of EBGM.

### 2.3 Time-to-onset analysis

TTO is characterized as the range between the time point when pregabalin is first used (START_DT field in the THER file) and the time point when AEs occur (EVENT_DT field in the DEMO file) (17). To ensure the accuracy of the analysis, this study excluded reports with input errors (e.g., EVENT_DT earlier than START_DT), unclear date records, or missing key data. We conducted a statistical analysis of TTO using the median, quartiles, and Weibull shape parameter (WSP). It was utilized to demonstrate the temporal trend of AE incidence. The distribution is determined by two fundamental parameters: the scale parameter (*α*) and the shape parameter (*β*). Additionally, Kaplan–Meier analysis was employed to assess variations in TTO among different age groups.

### 2.4 Sensitivity analysis

The primary analysis was based on case reports in which pregabalin was designated as the primary suspect drug after exclusion of reports containing the predefined medications recorded as secondary suspect or concomitant drugs. Sensitivity analyses were performed on the following subgroups: (1) cases further restricted to physician as reporters; (2) adults aged 18–64.9 years; and (3) older adults aged ≥65 years.

## 3 Results

### 3.1 Baseline characteristics of pregabalin-related reports

This study extracted a total of 18,823,249 deduplicated AE reports from the FAERS database (Q1 2005 to Q1 2025). Among these, 116,672 deduplicated case reports identified pregabalin as the primary suspect drug. After applying the predefined exclusion criteria for selected medications recorded as secondary suspect or concomitant drugs, 98,196 eligible individual case safety reports(ICSRs), containing 295,395 adverse-event records, were included in the final analysis. Following the exclusion of 10,603 patients with unknown gender, females accounted for the majority of cases (*N* = 58,808, 59.9%), compared to males (*N* = 28,785, 29.3%). Among reports with explicit age data, patients aged 18–64 years constituted the largest group (*N* = 34,351, 35.0%), followed by those aged ≥65 years (*N* = 23,489, 23.9%). Most reports were submitted by consumers (*N* = 56,248, 57.3%) and originated from the United States (75.6%). Among serious AEs linked to pregabalin, other significant medical events (85.9%) and hospitalization (9.0%) were most common. Less frequently reported outcomes were death (2.9%), life-threatening conditions (1.2%), disability (1.0%), and congenital anomaly (0.1%). Further details are presented in Table 2.

**Table 2:** Clinical characteristics of adverse events associated with pregabalin treatment based on the FAERS database.

	Pregabalin
Case reports	(*N* = 98196)
Sex	
Female	58808 (59.9%)
Male	28785 (29.3%)
Missing	10603 (10.8%)
Age (year)	
< 18	1165 (1.2%)
18～64.9	34351 (35.0%)
65～85	21140 (21.5%)
> 85	2349 (2.4%)
Missing	39191 (39.9%)
Reporter Type	
Consumer	56248 (57.3%)
Health Professional	3825 (3.9%)
Pharmacist	5215 (5.3%)
Physician	21787 (22.2%)
Missing	11121 (11.3%)
Reporter Country	
United States	74199 (75.6%)
United Kingdom	2363 (2.4%)
France	3363 (3.4%)
Japan	2751 (2.8%)
Other Country	15520 (15.8%)
Outcome	
Death	2855 (2.9%)
Disability	948 (1.0%)
Hospitalization	8802 (9.0%)
Life-Threatening	1210 (1.2%)
Congenital Anomaly	64 (0.1%)
Other	84317 (85.9%)

### 3.2 Disproportionality analysis

Statistical analyses at the SOC and PT levels revealed that AEs associated with pregabalin were distributed across 27 SOCs (Supplementary Table 2), among which 7 SOCs generated positive signals based on both ROR and BCPNN algorithms. Furthermore, “social circumstances” was the only SOC for which a signal was identified by all four algorithms. Among 5,234 PTs meeting the signal-detection criteria of at least one of the four algorithms, 279 (5.3%) simultaneously met the predefined criteria of all four algorithms (Figure 2). The subsequent analysis was focused on the 30 most frequently reported PTs, the details of which are presented in Table 3**.** The most commonly reported AEs were dizziness (*n* = 5929, ROR=2.56, PRR = 2.53, EBGM05 = 2.51, IC025 = 1.33), weight increased (*n* = 5515, ROR=5.48, PRR = 5.39, EBGM05 = 5.27, IC025 = 2.4), and somnolence (*n* = 5045, ROR=5.48, PRR = 5.41, EBGM05 = 5.29, IC025 = 2.4).

**Figure 2:**
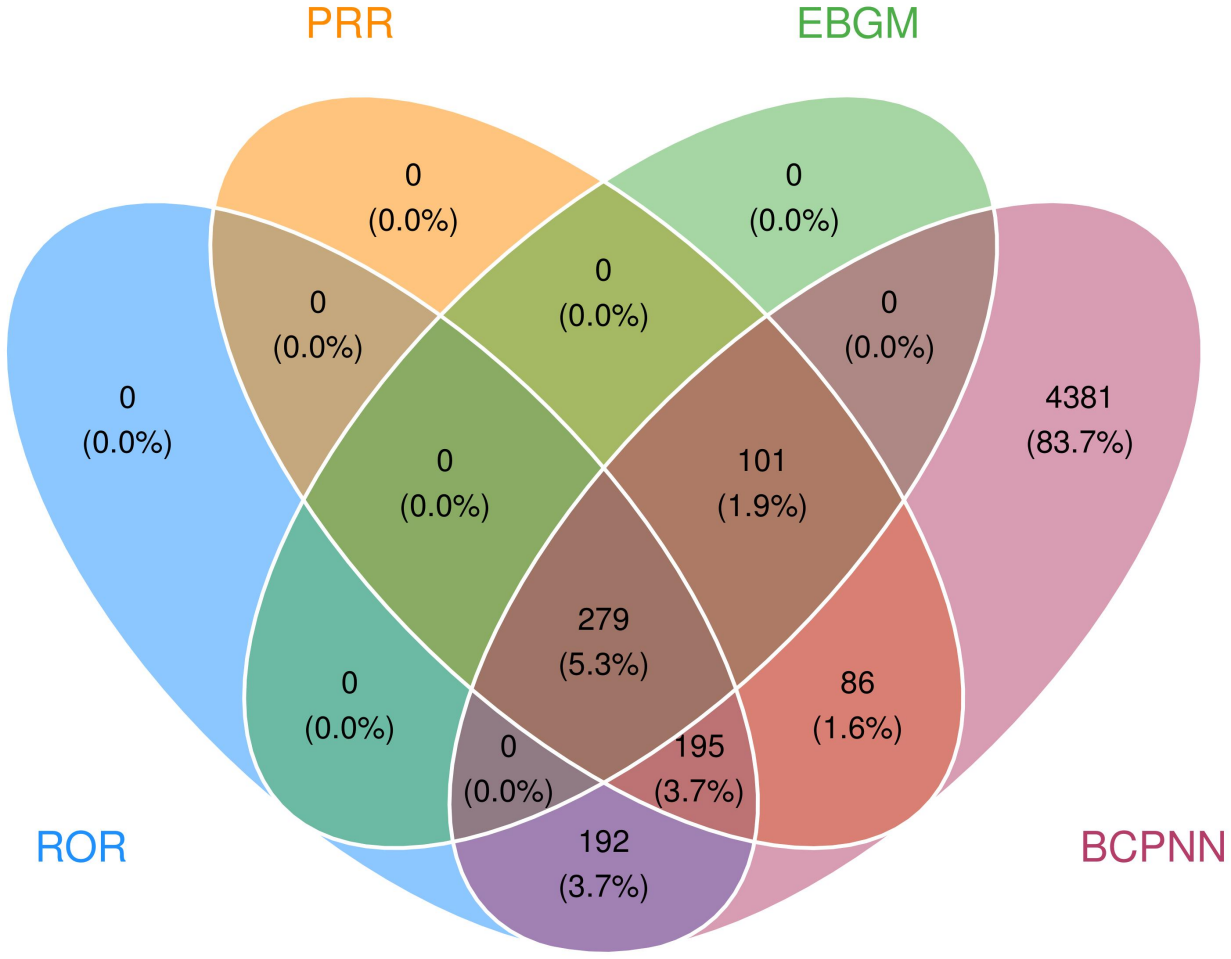
Venn diagram of significant pregabalin adverse event signals concurrently identified by four disproportionality methods.

**Table 3:** Top 30 adverse events of pregabalin in the FAERS database.

SOC	PT	N	ROR (95%Cl)	PRR(*χ*^2^)	EBGM(EBGM05)	IC(IC025)
Nervous system disorders	dizziness	5929	2.56 (2.49–2.62)	2.53 (5435.27)	2.51 (2.45)	1.33 (1.29)
Investigations	weight increased	5515	5.48 (5.33–5.63)	5.39 (19255.88)	5.27 (5.15)	2.4 (2.36)
Nervous system disorders	somnolence	5045	5.48 (5.33–5.64)	5.41 (17678.13)	5.29 (5.16)	2.4 (2.36)
General disorders and administration site conditions	malaise	4758	2.26 (2.19–2.32)	2.24 (3237.71)	2.22 (2.17)	1.15 (1.11)
General disorders and administration site conditions	feeling abnormal	4496	3.95 (3.84–4.07)	3.91 (9573.01)	3.85 (3.76)	1.94 (1.9)
Psychiatric disorders	insomnia	3687	2.92 (2.83–3.02)	2.9 (4538.87)	2.87 (2.79)	1.52 (1.47)
General disorders and administration site conditions	gait disturbance	3024	3.25 (3.13–3.37)	3.22 (4578.99)	3.19 (3.09)	1.67 (1.62)
Eye disorders	vision blurred	2981	4.76 (4.59–4.94)	4.72 (8551.91)	4.63 (4.49)	2.21 (2.16)
General disorders and administration site conditions	withdrawal syndrome	2863	15.05 (14.49–15.64)	14.91 (34479.09)	13.9 (13.46)	3.8 (3.74)
Nervous system disorders	memory impairment	2225	3.41 (3.26–3.55)	3.39 (3685.57)	3.35 (3.23)	1.74 (1.68)
General disorders and administration site conditions	oedema peripheral	2112	3.65 (3.49–3.81)	3.63 (3953.65)	3.58 (3.45)	1.84 (1.78)
Psychiatric disorders	confusional state	2046	2.69 (2.57–2.81)	2.67 (2121.2)	2.65 (2.56)	1.41 (1.34)
Nervous system disorders	tremor	1973	2.5 (2.39–2.62)	2.49 (1747.29)	2.47 (2.38)	1.31 (1.24)
Nervous system disorders	hypoaesthesia	1698	2.34 (2.23–2.46)	2.33 (1279.82)	2.32 (2.22)	1.21 (1.14)
Nervous system disorders	paraesthesia	1597	2.11 (2.01–2.22)	2.11 (921.27)	2.1 (2.01)	1.07 (0.99)
General disorders and administration site conditions	peripheral swelling	1586	2.19 (2.09–2.3)	2.19 (1010.47)	2.17 (2.08)	1.12 (1.05)
Nervous system disorders	burning sensation	1445	4.38 (4.16–4.62)	4.37 (3669.64)	4.29 (4.11)	2.1 (2.02)
General disorders and administration site conditions	drug withdrawal syndrome	1412	3.3 (3.13–3.47)	3.28 (2208.6)	3.25 (3.11)	1.7 (1.62)
General disorders and administration site conditions	swelling	1351	2.55 (2.41–2.69)	2.54 (1247.87)	2.52 (2.41)	1.33 (1.25)
Psychiatric disorders	suicidal ideation	1350	3.13 (2.97–3.3)	3.12 (1916.74)	3.09 (2.95)	1.63 (1.55)
Nervous system disorders	balance disorder	1308	3.17 (3–3.35)	3.16 (1905.32)	3.13 (2.99)	1.64 (1.56)
Eye disorders	visual impairment	1308	2.18 (2.06–2.3)	2.17 (822.39)	2.16 (2.06)	1.11 (1.03)
Gastrointestinal disorders	dry mouth	1131	3 (2.83–3.19)	3 (1481.92)	2.96 (2.82)	1.57 (1.48)
Ear and labyrinth disorders	hypoacusis	1039	4.51 (4.24–4.8)	4.5 (2764.59)	4.42 (4.2)	2.14 (2.05)
Nervous system disorders	amnesia	1038	3.33 (3.13–3.54)	3.33 (1660.54)	3.29 (3.12)	1.72 (1.63)
Nervous system disorders	disturbance in attention	1016	3.95 (3.72–4.21)	3.94 (2188.87)	3.88 (3.69)	1.96 (1.87)
Nervous system disorders	speech disorder	938	3.79 (3.55–4.04)	3.78 (1883.93)	3.73 (3.53)	1.9 (1.8)
General disorders and administration site conditions	gait inability	875	3.2 (2.99–3.42)	3.19 (1296.51)	3.16 (2.98)	1.66 (1.56)
Psychiatric disorders	agitation	766	2.2 (2.05–2.36)	2.19 (492.4)	2.18 (2.05)	1.12 (1.02)
Nervous system disorders	coma	637	2.92 (2.7–3.16)	2.92 (791.6)	2.89 (2.71)	1.53 (1.42)

SOC, System Organ Class; PT, Preferred Terms; N, number of adverse events exported; ROR, reporting odds ratio; PRR, proportional reporting ratio; 95% CI, 95% confidence interval; χ^2^, chi-squared; IC, information component; IC025, the lower limit of 95% CI of the IC; EBGM, empirical Bayesian geometric mean; EBGM05, the lower limit of 95% CI of EBGM.

When ordered according to ROR signal strength, the five most prominent AEs were withdrawal syndrome (*n* = 2863, ROR=15.05, PRR = 14.91, EBGM05 = 13.9, IC025 = 3.8), somnolence (*n* = 5045, ROR=5.48, PRR = 5.41, EBGM05 = 5.29, IC025 = 2.4), weight increased (*n* = 5515, ROR=5.48, PRR = 5.39, EBGM05 = 5.27, IC025 = 2.4), vision blurred (*n* = 2981, ROR=4.76, PRR = 4.72, EBGM05 = 4.63, IC025 = 2.21), and hypoacusis (*n* = 1039, ROR=4.51, PRR = 4.5, EBGM05 = 4.42, IC025 = 2.14). Our analysis identified several unexpected AEs associated with pregabalin, including hypoacusis, dysgraphia, logorrhoea, and feeling of despair. Furthermore, events described as rare in the prescribing information, such as coma and dyskinesia, were reported more frequently than expected in this real-world cohort. Key results are shown in Supplementary Table 4.

### 3.3 Stratified analysis

Comparison of age subgroups revealed that older adults (≥65 years) faced an increased risk for falls (OR=2.26, *P* < 0.001), balance disorder (OR=1.91, *P* < 0.001), and confusional state (OR=1.81, *P* < 0.001). In contrast, adults (18–64.9 years) more frequently reported withdrawal syndrome (OR=0.54, *P* < 0.001), drug dependence (OR=0.46, *P* < 0.001), and suicidal ideation (OR=0.38, *P* < 0.001). Key results are shown in Table 4.

**Table 4:** Comparative analysis of adverse event reporting for pregabalin between older adults (≥65 years) and adults (18–64.9 years) in the FAERS database.

PT	Age		OR (95%CI)	*P*-value
	≥65 years (*N* = 81474)	18–64.9 years (*N* = 1,24,001)		
weight increased	1054	2051	0.78 (0.72–0.84)	＜0.001
malaise	1200	1946	0.94 (0.87–1.01)	0.08
dizziness	1910	1906	1.54 (1.44–1.64)	＜0.001
feeling abnormal	1133	1892	0.91 (0.85–0.98)	＜0.05
somnolence	1453	1778	1.25 (1.16–1.34)	＜0.001
insomnia	1073	1620	1.01 (0.93–1.09)	0.84
withdrawal syndrome	541	1528	0.54 (0.49–0.59)	＜0.001
headache	582	1404	0.63 (0.57–0.69)	＜0.001
nausea	694	1297	0.81 (0.74–0.89)	＜0.001
fatigue	710	1246	0.87 (0.79–0.95)	＜0.05
gait disturbance	1088	1173	1.42 (1.3–1.54)	＜0.001
vision blurred	771	1026	1.15 (1.04–1.26)	＜0.05
anxiety	361	1024	0.53 (0.47–0.6)	＜0.001
memory impairment	738	920	1.22 (1.11–1.35)	＜0.001
fall	1343	913	2.26 (2.08–2.46)	＜0.001
drug hypersensitivity	500	875	0.87 (0.78–0.97)	＜0.05
drug dependence	266	868	0.46 (0.4–0.53)	＜0.001
depression	311	782	0.6 (0.53–0.69)	＜0.001
tremor	629	769	1.25 (1.12–1.39)	＜0.001
hypoaesthesia	435	741	0.89 (0.79–1.01)	0.06
paraesthesia	391	732	0.81 (0.72–0.92)	＜0.001
dyspnoea	541	726	1.14 (1.02–1.27)	＜0.05
drug withdrawal syndrome	202	699	0.44 (0.37–0.51)	＜0.001
confusional state	817	691	1.81 (1.63–2)	＜0.001
suicidal ideation	169	683	0.38 (0.32–0.44)	＜0.001
vomiting	323	668	0.73 (0.64–0.84)	＜0.001
diarrhoea	362	640	0.86 (0.76–0.98)	＜0.05
oedema peripheral	490	616	1.21 (1.08–1.37)	＜0.05
arthralgia	362	614	0.9 (0.79–1.02)	0.1
burning sensation	425	603	1.07 (0.95–1.22)	0.27
peripheral swelling	520	579	1.37 (1.22–1.54)	＜0.001
seizure	198	568	0.53 (0.45–0.62)	＜0.001
hyperhidrosis	209	552	0.58 (0.49–0.67)	＜0.001
weight decreased	375	552	1.03 (0.91–1.18)	0.62
asthenia	559	549	1.55 (1.38–1.75)	＜0.001
pruritus	402	541	1.13 (0.99–1.29)	0.06
back pain	341	538	0.96 (0.84–1.1)	0.6
muscle spasms	315	528	0.91 (0.79–1.04)	0.17
visual impairment	412	503	1.25 (1.1–1.42)	＜0.001
swelling	275	472	0.89 (0.76–1.03)	0.11
loss of consciousness	343	458	1.14 (0.99–1.31)	0.07
rash	345	453	1.16 (1.01–1.33)	＜0.05
amnesia	229	448	0.78 (0.66–0.91)	＜0.05
disturbance in attention	236	446	0.8 (0.69–0.94)	＜0.05
neuralgia	198	411	0.73 (0.62–0.87)	＜0.001
agitation	131	411	0.48 (0.4–0.59)	＜0.001
nasopharyngitis	207	405	0.78 (0.66–0.92)	＜0.05
balance disorder	501	401	1.91 (1.67–2.18)	＜0.001
neuropathy peripheral	325	399	1.24 (1.07–1.44)	＜0.05
dry mouth	354	398	1.36 (1.17–1.56)	＜0.001

The listed adverse events had significant signal strengths, as assessed by odds ratios (OR) with 95% confidence intervals.

All analyses were conducted using the Pearson chi-square test.

*P*-value < 0.05 were considered statistically significant.

### 3.4 Time-to-onset analysis

Following the removal of reports containing incomplete data, ambiguous, or undisclosed onset times, 8,538 reports were included in the TTO analysis. Among them, 3,990 (46.7%) were adults (18–64.9 years) and 3,264 (38.2%) from the older adults (≥65 years). As shown in Figure 3, The blue-green bars represent the number of reports in each interval, whereas the red bars represent the corresponding percentage of the total reports included in the TTO analysis. Overall, 54.53% of AEs occurred within the first 30 days after treatment initiation, with a gradual decline thereafter. The median TTO was shorter in older adults (16 days, IQR: 3–90) compared to adults (30 days, IQR: 5–151, Table 5). The Weibull shape parameters (*β*) for both groups were below 1 (0.46 and 0.48, respectively), indicating an early failure pattern. Kaplan–Meier analysis confirmed earlier AEs in the elderly (Figure 4, Log-rank *P* < 0.001), supporting an early-risk profile for pregabalin-associated AEs, particularly in older adults.

**Figure 3:**
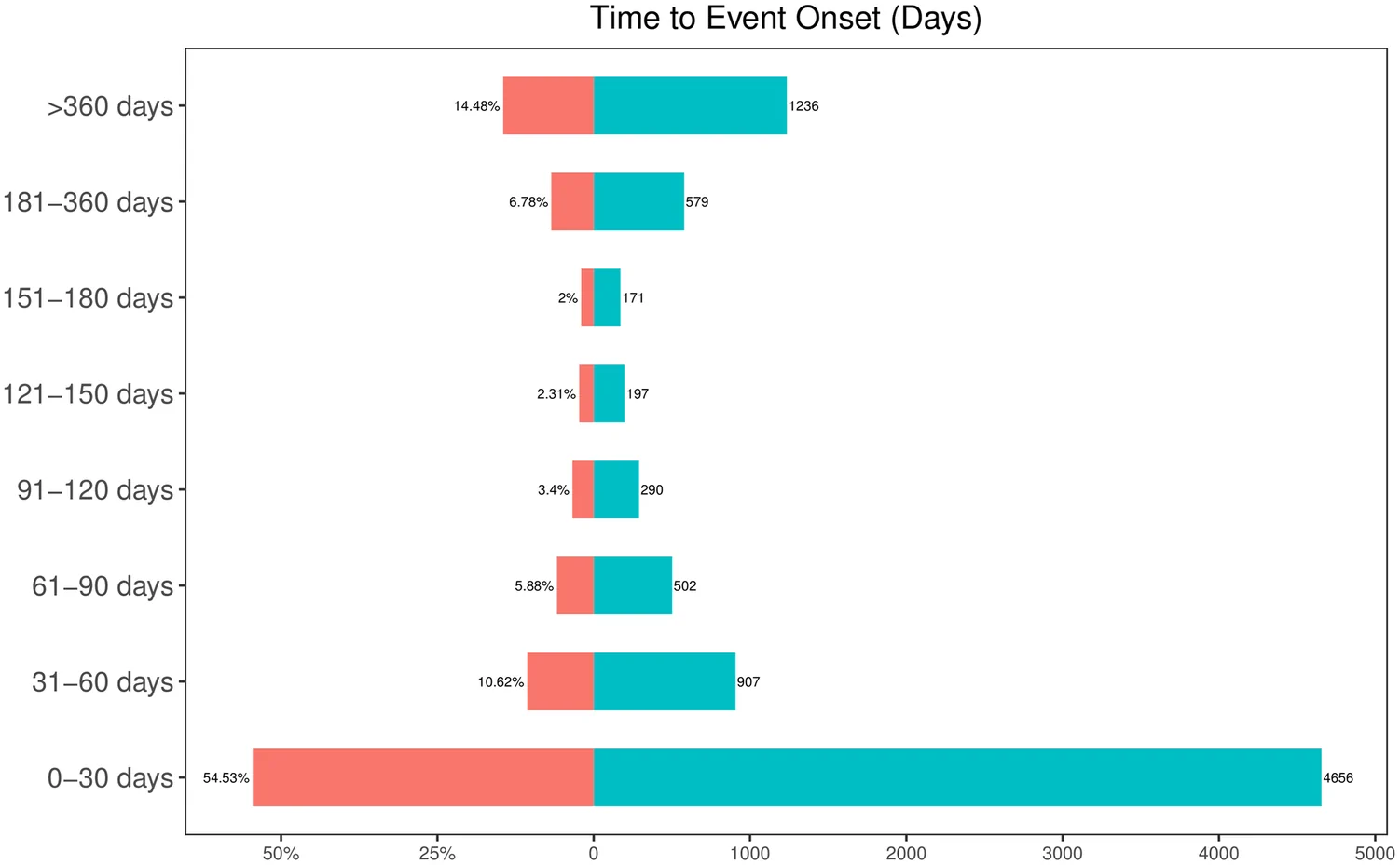
Onset time distribution of adverse events associated with pregabalin. The blue-green bars on the right indicate the number of reports in each time interval, whereas the red bars on the left indicate the corresponding percentage of total reports included in the time-to-onset analysis. The largest proportion of adverse events occurred within 0−30 days after pregabalin initiation.

**Table 5:** Time-to-onset analysis of adverse events.

	TTO (days)			Weibull Distribution Test				Failure Type
Group	N	Median (IQR)	Min-Max	*α*	95% CI	*β*	95% CI	
Overall	8538	24 (4–123)	1–8630	73.65	70.14–77.16	0.47	0.47–0.48	Early failure
Older adults (≥65 years)	3264	16 (3–90)	1–5508	58.67	54.05–63.28	0.46	0.45–0.48	Early failure
Adults (18–64.9 years)	3990	30 (5–151)	1–8630	86.08	80.19–91.98	0.48	0.47–0.49	Early failure

TTO, Time-to-onset; N, number of adverse event reports; IQR, interquartile range; CI, Confidence Interval; α, scale parameter; β, shape parameter.

**Figure 4:**
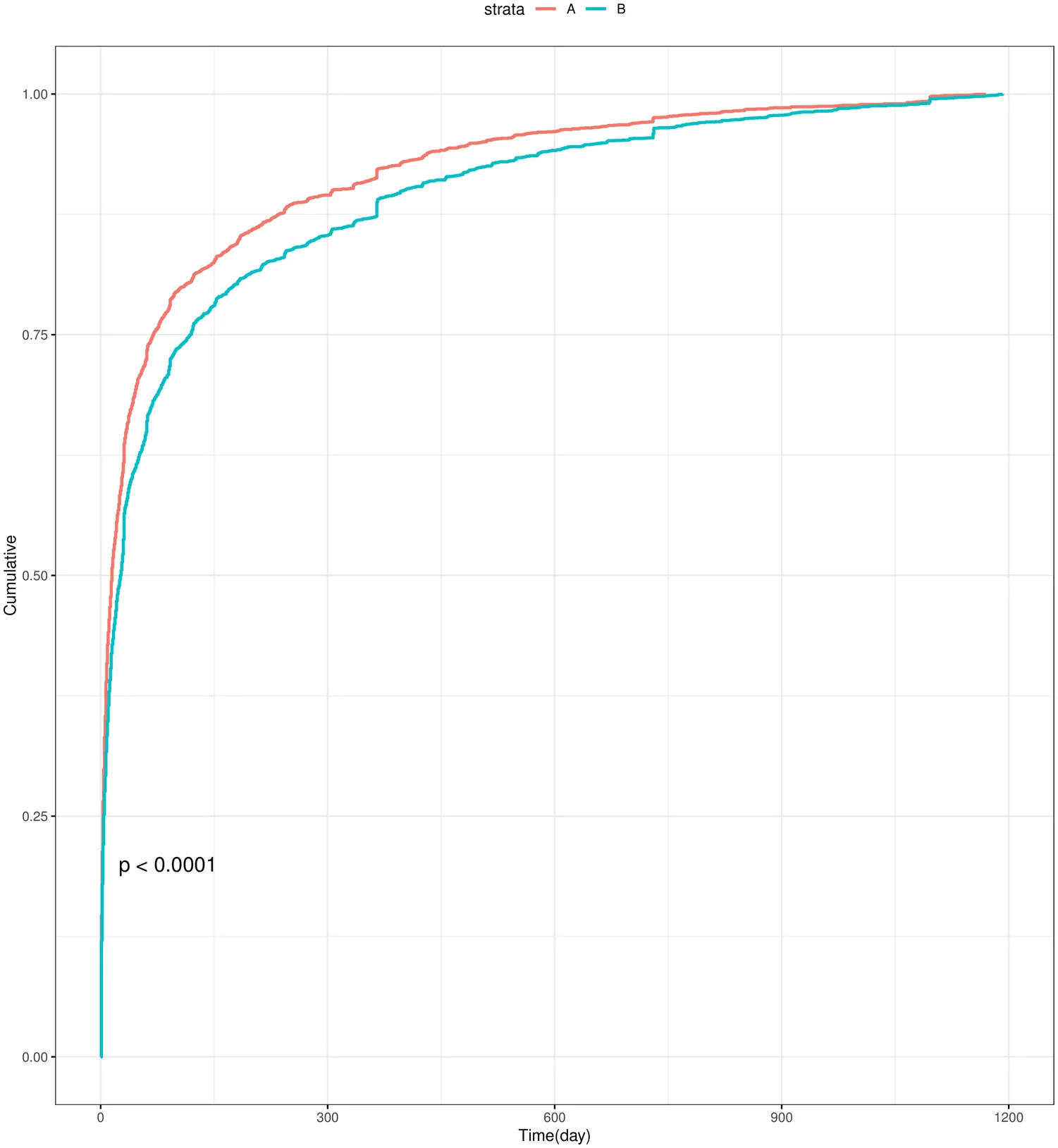
Cumulative incidence of pregabalin-associated adverse events by age group. A significant difference was observed between Group A (≥65 years) and Group B (18–64.9 years) (log-rank test, *p* < 0.0001).

### 3.5 Sensitivity analysis

The primary analysis included 98,196 eligible pregabalin-related ICSRs after exclusion of reports involving the selected concomitant medications. Three sensitivity analyses were performed on this analytic dataset: stratification by age (34,351 reports involving individuals aged 18–64.9 years and 23,489 reports involving individuals aged ≥65 years) and restriction to case reports submitted exclusively by physicians (21,787 physician-reported ICSRs). The 30 primary pregabalin signals showed high robustness in sensitivity analyses, with all signals remaining significant across age and physician-reported subgroups. Specific PTs, including withdrawal syndrome and confusional state, displayed higher ROR estimates in the elderly, while memory impairment and hypoacusis demonstrated stronger associations in physician-reported cases. No new signals were identified. Detailed results are included in Supplementary Table 3.

## 4 Discussion

This pharmacovigilance analysis leveraged the FAERS database to evaluate the post-marketing safety of pregabalin. Our research identified that dizziness, somnolence, and weight gain were the most frequently reported AEs to pregabalin, in line with results reported in prior clinical studies. In a one-year study of patients with anxiety disorders, dizziness (12.5%), somnolence (7.6%), and weight gain (5.5%) were likewise identified as the most common treatment-related AEs (18). By selectively binding to the *α*₂*δ* subunit of voltage-gated calcium channels in the central nervous system (CNS), pregabalin reduces the release of excitatory neurotransmitters. This central inhibition is the key mechanism behind both its therapeutic effects and the dose-limiting CNS-mediated side effects, such as somnolence (19). Pregabalin also exhibits a high density of binding sites in the cerebellum. Its inhibitory effect on neuronal excitability is proposed to disrupt normal cerebellar function, contributing to dizziness and dyskinesia (20). Furthermore, the weight gain associated with pregabalin is dose- and duration-dependent, as supported by a meta-analysis indicating that most weight changes occur within ±7% of baseline body weight (21).

Withdrawal syndrome represents a clinically significant risk associated with pregabalin therapy. While the risk is low when tapering gradually (≥1 week) (22), it may be triggered in real-world scenarios by factors such as abrupt discontinuation, rapid dose reduction, prolonged high-dose use, and substance misuse (23).

Coma and dyskinesia, which are designated as rare events in the prescribing information, were reported more frequently than expected in this real-world cohort. Pregabalin exerts its effects by blocking voltage-dependent calcium channels, reducing excitatory neurotransmitter release. When used in excessive amounts, it may cause significant central nervous system depression (24). There have been case reports of a patient with mild kidney dysfunction developing coma after concomitant use of baclofen and pregabalin. Normal consciousness was restored after discontinuing the drugs (25). Although the incidence of coma is relatively low, the risk increases significantly with high-dose use, renal insufficiency, or concurrent use of other central nervous system depressants. Similarly, pregabalin-associated dyskinesia warrants clinical vigilance due to its potential to markedly impair daily functioning and quality of life. Published studies have documented a spectrum of pregabalin-related movement disorders, including myoclonus, Parkinson-like symptoms, and akathisia (26).

Hypoacusis and dysgraphia were identified as unexpected AE signals associated with pregabalin in our analysis. This observation aligns with earlier case studies, including one in which a patient with neuralgia developed significant hearing impairment after starting pregabalin at 450 mg daily. The symptoms improved upon reducing the dose to 300 mg daily, indicating that these adverse effects may be dose-dependent and reversible (27). Pregabalin may cause ataxia and tremor, leading to significant impairment of motor coordination and stability. Clinically, these effects are often observed as impaired fine motor skills, such as dysgraphia and difficulty grasping objects (28).

Age-stratified analysis indicated that falls and balance disorder were more frequently reported in older adults (≥65 years). A previous study using the FAERS database and the Japanese Adverse Drug Event Report (JADER) database also assessed fall-related AEs associated with pregabalin. That study found that pregabalin-associated fall-related AEs were reported more frequently in older adults (≥60 years) than in middle-aged and younger populations (29). Furthermore, A Japanese pharmacoepidemiological study identified pregabalin use as a significant risk factor for fractures, particularly in elderly patients (30). Pregabalin may contribute to balance disorder and increased falls risk through multiple mechanisms, including sensory neuropathy, cerebellar dysfunction, vestibular impairment, cognitive effects, and blood pressure dysregulation (31). Therefore, when prescribing pregabalin to older adults, the potential risks should be carefully considered. In contrast, young adults (aged 18–64.9 years) more frequently reported suicidal ideation. However, these findings should be interpreted cautiously, as pregabalin is commonly used to treat chronic neuropathic pain and anxiety disorders—conditions that are themselves significant risk factors for suicidal ideation, making it challenging to definitively attribute the source in clinical practice (32).

The TTO analysis revealed an early failure pattern for pregabalin-associated AEs, with more than half occurring during the initial month of therapy. This trend was especially pronounced in older adults, who experienced both earlier onset and a higher cumulative risk. The age-related differences observed are probably attributable to reductions in liver and kidney function, which impair drug clearance and promote accumulation (33). Therefore, safety monitoring during the initial stage of clinical use, particularly within the first month, is recommended, with enhanced vigilance warranted for older adults.

This study has several limitations. As a spontaneous reporting database, FAERS is subject to underreporting, incomplete information, selective or stimulated reporting, and residual confounding. Because reliable exposure denominators are unavailable, incidence and absolute risk cannot be estimated. Reporting patterns may also differ across age groups because of differences in comorbidities, polypharmacy, healthcare utilization, and clinical surveillance. In addition, excluding selected secondary suspect or concomitant medications may have introduced selection bias and reduced real-world representativeness. Therefore, the findings should be interpreted as reporting signals rather than causal associations.

## 5 Conclusion

This pharmacovigilance study systematically evaluated pregabalin's safety using multiple disproportionality analyses of FAERS data. Most of the detected signals were consistent with the drug label information, while unexpected signals, including hypoacusis and dysgraphia, were also identified. Age-stratified analysis revealed higher relative risks for falls and balance disorders in older adults (≥65 years), whereas withdrawal syndrome, drug dependence, and suicidal ideation were more common in adults (18–64.9 years). TTO analysis showed that over half of the AEs occurred within the first month of treatment, with older adults (≥65 years) exhibiting earlier onset and a higher cumulative risk. Therefore, safety monitoring during the initial stage of clinical use, particularly within the first month, is recommended, with enhanced vigilance warranted for older adults. Our findings enhance the current understanding of pregabalin's safety profile, although these novel signals require confirmation through additional mechanistic studies.

## Data Availability

The original contributions presented in the study are included in the article/Supplementary Material, further inquiries can be directed to the corresponding authors.
