# Supplementary material for: Age-stratified signals and early onset of adverse events with pregabalin: a disproportionality analysis of the FDA adverse event reporting system
Source: Front Med (Lausanne). 2026 Oct 1;13:1806099. doi: 10.3389/fmed.2026.1806099 (PMC13668812; doi:10.3389/fmed.2026.1806099)
Supplement: Supplementary file 1 [file Supplementaryfile1.docx]

# Supplementary Table S1. READUS-PV Checklist

**Manuscript title:** Age-stratified adverse event reporting patterns associated with pregabalin: a disproportionality analysis of the FDA Adverse Event Reporting System

| READUS-PV item | Reported in manuscript | Location in manuscript |
| --- | --- | --- |
| Title identifies study as disproportionality analysis and names database | Yes | Title |
| Structured abstract with objective, methods, results and conclusion | Yes | Abstract |
| Scientific background and rationale | Yes | Introduction, paragraphs 1–2 |
| Study objectives | Yes | Final paragraph of Introduction |
| Study design | Yes | Methods 2.1–2.2 |
| Data source (FAERS), study period and coding system | Yes | Methods 2.1 |
| Drug definition (Primary Suspect; generic/brand name) | Yes | Methods 2.1 |
| Data cleaning and duplicate removal | Yes | Methods 2.1 |
| Outcome definition (MedDRA PT/SOC) | Yes | Methods 2.1 |
| Statistical methods and signal detection algorithms | Yes | Methods 2.2; Table 1 |
| Signal thresholds | Yes | Methods 2.2; Table 1 |
| Age-stratified analysis | Yes | Methods 2.2 |
| Time-to-onset analysis | Yes | Methods 2.3 |
| Sensitivity analysis | Yes | Methods 2.4 |
| Software | Yes | Methods 2.2 |
| Study flow and included/excluded reports | Yes | Figure 1; Results |
| Baseline characteristics | Yes | Results; Table 2 |
| Disproportionality results | Yes | Results; Tables 3–4 |
| Discussion in context of previous literature | Yes | Discussion |
| Interpretation avoids causal inference | Yes | Methods 2.2; Discussion; Conclusion |
| Limitations of spontaneous reporting system | Yes | Discussion (Limitations) |
| Funding | Yes | Funding |
| Conflict of interest | Yes | Conflict of Interest |
| Ethics | Yes | Ethics approval |
| Data availability | Yes | Data Availability Statement |

Note: This checklist was completed according to the READUS-PV reporting recommendations. Locations refer to manuscript sections in the revised version rather than final typeset page numbers.
