# Supplementary material for: Age-stratified signals and early onset of adverse events with pregabalin: a disproportionality analysis of the FDA adverse event reporting system
Source: Front Med (Lausanne). 2026 Oct 1;13:1806099. doi: 10.3389/fmed.2026.1806099 (PMC13668812; doi:10.3389/fmed.2026.1806099)
Supplement: Supplementary file 2 [file Supplementaryfile2.docx]

**Supplementary Table 1. 2 × 2 fourfold table of disproportionality method.**

|  | Pregabalin-related ADEs | Non-pregabalin-related ADEs | Total |
| --- | --- | --- | --- |
| Pregabalin | a | b | a + b |
| Non-pregabalin | c | d | c + d |
| Total | a + c | b + d | N = a + b + c + d |

**Abbreviations: ADEs, adverse drug events；N, the number of reports.**

**Supplementary Table 2. SOC-level disproportionality analysis of pregabalin-related adverse events in the FAERS database.**

| SOC | N | ROR (95%Cl) | PRR(χ²) | EBGM(EBGM05) | IC(IC025) |
| --- | --- | --- | --- | --- | --- |
| General disorders and administration site conditions | 70364 | 1.48 (1.47 - 1.49) | 1.37 (8291.56) | 1.36 (1.35) | 0.45 (0.43) |
| Musculoskeletal and connective tissue disorders | 19650 | 1.3 (1.28 - 1.32) | 1.28 (1233.41) | 1.27 (1.26) | 0.35 (0.33) |
| Investigations | 15082 | 0.83 (0.82 - 0.85) | 0.84 (484.77) | 0.84 (0.83) | -0.25 ( -0.27) |
| Eye disorders | 9658 | 1.67 (1.64 - 1.7) | 1.65 (2488.7) | 1.64 (1.61) | 0.72 (0.69) |
| Injury, poisoning and procedural complications | 26819 | 0.85 (0.84 - 0.86) | 0.86 (670.46) | 0.86 (0.85) | -0.21 ( -0.23) |
| Nervous system disorders | 48703 | 2.16 (2.14 - 2.18) | 1.97 (25159.22) | 1.96 (1.94) | 0.97 (0.96) |
| Psychiatric disorders | 32117 | 2.08 (2.05 - 2.1) | 1.96 (15849.87) | 1.95 (1.93) | 0.96 (0.95) |
| Vascular disorders | 3521 | 0.56 (0.54 - 0.58) | 0.56 (1227.5) | 0.56 (0.55) | -0.83 ( -0.88) |
| Gastrointestinal disorders | 15786 | 0.61 (0.6 - 0.62) | 0.63 (3799.87) | 0.63 (0.62) | -0.67 (-0.69) |
| Social circumstances | 3086 | 2.27 (2.19 - 2.35) | 2.26 (2149.1) | 2.24 (2.18) | 1.17 (1.11) |
| Metabolism and nutrition disorders | 4578 | 0.71 (0.69 - 0.73) | 0.72 (518.81) | 0.72 (0.7) | -0.48 (-0.52) |
| Cardiac disorders | 4844 | 0.63 (0.61 - 0.65) | 0.63 (1049.77) | 0.63 (0.62) | -0.66 ( -0.7) |
| Skin and subcutaneous tissue disorders | 9425 | 0.57 (0.56 - 0.59) | 0.59 (2883.5) | 0.59 (0.58) | -0.77 ( -0.8) |
| Immune system disorders | 3161 | 0.96 (0.93 - 1) | 0.96 (4.76) | 0.96 (0.93) | -0.06 ( -0.11) |
| Blood and lymphatic system disorders | 1293 | 0.25 (0.24 - 0.27) | 0.26 (2800.12) | 0.26 (0.25) | -1.95 ( -2.03) |
| Renal and urinary disorders | 3285 | 0.58 (0.56 - 0.6) | 0.59 (965.64) | 0.59 (0.57) | -0.76 ( -0.81) |
| Infections and infestations | 6620 | 0.41 (0.4 - 0.42) | 0.42 (5492.11) | 0.42 (0.42) | -1.24 ( -1.27) |
| Reproductive system and breast disorders | 1251 | 0.49 (0.46 - 0.51) | 0.49 (679.19) | 0.49 (0.47) | -1.03 (-1.12) |
| Neoplasms benign, malignant and unspecified (incl cysts and polyps) | 1978 | 0.25 (0.24 - 0.26) | 0.26 (4387.58) | 0.26 (0.25) | -1.96 (-2.03) |
| Ear and labyrinth disorders | 2427 | 1.92 (1.84 - 2) | 1.91 (1047.95) | 1.9 (1.84) | 0.93 (0.87) |
| Respiratory, thoracic and mediastinal disorders | 7966 | 0.56 (0.55 - 0.57) | 0.57 (2660.15) | 0.57 (0.56) | -0.8 (-0.83) |
| Product issues | 781 | 0.16 (0.15 - 0.17) | 0.16 (3491.76) | 0.16 (0.15) | -2.64 (-2.74) |
| Hepatobiliary disorders | 1347 | 0.5 (0.47 - 0.52) | 0.5 (681.12) | 0.5 (0.48) | -1 (-1.08) |
| Pregnancy, puerperium and perinatal conditions | 213 | 0.17 (0.15 - 0.19) | 0.17 (863.3) | 0.17 (0.15) | -2.55 (-2.74) |
| Endocrine disorders | 401 | 0.53 (0.48 - 0.58) | 0.53 (168.26) | 0.53 (0.49) | -0.92 (-1.06) |
| Congenital, familial and genetic disorders | 262 | 0.28 (0.25 - 0.32) | 0.28 (475.8) | 0.28 (0.26) | -1.81 (-1.99) |
| Surgical and medical procedures | 777 | 0.19 (0.18 - 0.2) | 0.19 (2665.89) | 0.19 (0.18) | -2.37 (-2.47) |

**Abbreviations: SOC, System Organ Class; N, number of adverse events exported; ROR, reporting odds ratio; CI, confidence interval; PRR, proportional reporting ratio; χ², chi-square; EBGM, empirical Bayesian geometric mean; EBGM05, the lower 95% one-sided confidence limit of EBGM; IC, information component; IC025, the lower limit of the 95% credibility interval for IC.**

**Supplementary Table 3. Safety signals for pregabalin-associated adverse events in the FAERS database: primary and sensitivity analyses.**

| Primary Suspect Group | | | Subgroup: 18-64.9 years | | Subgroup: ≥65 years | | Subgroup: Physician Reports | |
| --- | --- | --- | --- | --- | --- | --- | --- | --- |
| PT | case reports | ROR (95%CI) | case reports | ROR (95%CI) | case reports | ROR (95%CI) | case reports | ROR (95%CI) |
| dizziness | 5929 | 2.56 (2.49 - 2.62) | 1906 | 1.88 (1.79 - 1.96) | 1910 | 2.58 (2.47 - 2.71) | 1143 | 3.13 (2.95 - 3.32) |
| weight increased | 5515 | 5.48 (5.33 - 5.63) | 2051 | 4.43 (4.23 - 4.63) | 1054 | 5.89 (5.54 - 6.26) | 908 | 5.48 (5.12 - 5.85) |
| somnolence | 5045 | 5.48 (5.33 - 5.64) | 1778 | 4.62 (4.41 - 4.84) | 1453 | 5.93 (5.62 - 6.25) | 1037 | 6.24 (5.86 - 6.64) |
| malaise | 4758 | 2.26 (2.19 - 2.32) | 1946 | 2.3 (2.2 - 2.41) | 1200 | 1.93 (1.82 - 2.04) | 721 | 2.05 (1.91 - 2.21) |
| feeling abnormal | 4496 | 3.95 (3.84 - 4.07) | 1892 | 3.85 (3.68 - 4.03) | 1133 | 4.14 (3.9 - 4.4) | 644 | 4.77 (4.41 - 5.16) |
| insomnia | 3687 | 2.92 (2.83 - 3.02) | 1620 | 2.75 (2.61 - 2.88) | 1073 | 3.63 (3.41 - 3.86) | 581 | 3.27 (3.01 - 3.55) |
| gait disturbance | 3024 | 3.25 (3.13 - 3.37) | 1173 | 3.09 (2.91 - 3.27) | 1088 | 3.52 (3.31 - 3.74) | 544 | 3.57 (3.28 - 3.89) |
| vision blurred | 2981 | 4.76 (4.59 - 4.94) | 1026 | 3.58 (3.36 - 3.81) | 771 | 4.69 (4.36 - 5.04) | 447 | 4.1 (3.74 - 4.51) |
| withdrawal syndrome | 2863 | 15.05 (14.49 - 15.64) | 1528 | 18.79 (17.82 - 19.81) | 541 | 27.51 (25.09 - 30.16) | 618 | 24.49 (22.52 - 26.64) |
| memory impairment | 2225 | 3.41 (3.26 - 3.55) | 920 | 3.41 (3.2 - 3.64) | 738 | 4.38 (4.07 - 4.72) | 422 | 5.13 (4.66 - 5.66) |
| oedema peripheral | 2112 | 3.65 (3.49 - 3.81) | 616 | 2.59 (2.39 - 2.8) | 490 | 2.03 (1.85 - 2.22) | 547 | 3.46 (3.18 - 3.77) |
| confusional state | 2046 | 2.69 (2.57 - 2.81) | 691 | 2.38 (2.21 - 2.57) | 817 | 2.49 (2.32 - 2.67) | 539 | 3.19 (2.93 - 3.48) |
| tremor | 1973 | 2.5 (2.39 - 2.62) | 769 | 2.22 (2.07 - 2.38) | 629 | 2.72 (2.51 - 2.94) | 413 | 3.2 (2.91 - 3.53) |
| hypoaesthesia | 1698 | 2.34 (2.23 - 2.46) | 741 | 1.84 (1.72 - 1.98) | 435 | 2.92 (2.65 - 3.21) | 301 | 2.37 (2.11 - 2.65) |
| paraesthesia | 1597 | 2.11 (2.01 - 2.22) | 732 | 1.71 (1.59 - 1.84) | 391 | 2.7 (2.45 - 2.99) | 269 | 1.95 (1.73 - 2.2) |
| peripheral swelling | 1586 | 2.19 (2.09 - 2.3) | 579 | 2.06 (1.89 - 2.23) | 520 | 1.98 (1.81 - 2.16) | 253 | 2.24 (1.98 - 2.54) |
| burning sensation | 1445 | 4.38 (4.16 - 4.62) | 603 | 3.81 (3.51 - 4.13) | 425 | 5.41 (4.91 - 5.96) | 219 | 4.83 (4.22 - 5.52) |
| drug withdrawal syndrome | 1412 | 3.3 (3.13 - 3.47) | 699 | 6.03 (5.59 - 6.5) | 202 | 10.51 (9.11 - 12.12) | 229 | 4.54 (3.98 - 5.18) |
| swelling | 1351 | 2.55 (2.41 - 2.69) | 472 | 1.87 (1.71 - 2.05) | 275 | 2.12 (1.88 - 2.39) | 175 | 1.78 (1.53 - 2.07) |
| suicidal ideation | 1350 | 3.13 (2.97 - 3.3) | 683 | 2.96 (2.74 - 3.19) | 169 | 4.68 (4.01 - 5.45) | 282 | 3.21 (2.85 - 3.61) |
| balance disorder | 1308 | 3.17 (3 - 3.35) | 401 | 2.43 (2.2 - 2.68) | 501 | 3.38 (3.09 - 3.69) | 232 | 3.83 (3.36 - 4.36) |
| visual impairment | 1308 | 2.18 (2.06 - 2.3) | 503 | 2.23 (2.04 - 2.43) | 412 | 2.42 (2.19 - 2.67) | 267 | 2.92 (2.59 - 3.3) |
| dry mouth | 1131 | 3 (2.83 - 3.19) | 398 | 2.57 (2.33 - 2.84) | 354 | 2.91 (2.62 - 3.23) | 166 | 3.19 (2.74 - 3.72) |
| hypoacusis | 1039 | 4.51 (4.24 - 4.8) | 222 | 5.08 (4.44 - 5.8) | 594 | 5.96 (5.49 - 6.47) | 229 | 7.82 (6.85 - 8.93) |
| amnesia | 1038 | 3.33 (3.13 - 3.54) | 448 | 3.16 (2.88 - 3.47) | 229 | 3.04 (2.67 - 3.47) | 219 | 4.78 (4.18 - 5.47) |
| disturbance in attention | 1016 | 3.95 (3.72 - 4.21) | 446 | 3.34 (3.04 - 3.67) | 236 | 5.65 (4.96 - 6.43) | 159 | 3.6 (3.08 - 4.22) |
| speech disorder | 938 | 3.79 (3.55 - 4.04) | 390 | 3.66 (3.31 - 4.05) | 276 | 3.59 (3.19 - 4.05) | 187 | 4.38 (3.79 - 5.07) |
| gait inability | 875 | 3.2 (2.99 - 3.42) | 321 | 2.89 (2.59 - 3.23) | 287 | 3.19 (2.83 - 3.58) | 156 | 4.19 (3.58 - 4.91) |
| agitation | 766 | 2.2 (2.05 - 2.36) | 411 | 2.49 (2.26 - 2.75) | 131 | 1.65 (1.39 - 1.96) | 237 | 2.91 (2.56 - 3.31) |
| coma | 637 | 2.92 (2.7 - 3.16) | 377 | 3.06 (2.76 - 3.39) | 85 | 1.45 (1.17 - 1.79) | 351 | 5.09 (4.58 - 5.67) |

**Abbreviations: PT, Preferred Term; CI, confidence interval; ROR, reporting odds ratio.**

**Notes:**

**The primary cohort included reports with pregabalin as the sole suspected drug, after excluding those involving common concomitant medications. Sensitivity analyses were conducted by stratifying the primary cohort into age subgroups (18–64.9 years and ≥65 years) and by restricting to reports submitted exclusively by physicians.**

**A significant disproportionality signal was defined as a case count ≥ 3 and the lower limit of the 95% confidence interval (CI) for the ROR exceeding 1.**

**Supplementary Table 4. Unexpected and rare Safety Signals of Pregabalin in the FAERS Database.**

| PT | N | ROR (95%Cl) | PRR(χ²) | EBGM(EBGM05) | IC(IC025) |
| --- | --- | --- | --- | --- | --- |
| hypoacusis | 1039 | 4.51 (4.24 - 4.8) | 4.5 (2764.59) | 4.42 (4.2) | 2.14 (2.05) |
| dysgraphia | 213 | 6.81 (5.94 - 7.81) | 6.81 (1019.23) | 6.61 (5.89) | 2.72 (2.52) |
| logorrhoea | 75 | 4.47 (3.55 - 5.62) | 4.47 (197.08) | 4.39 (3.62) | 2.13 (1.8) |
| feeling of despair | 71 | 3.25 (2.57 - 4.12) | 3.25 (109) | 3.22 (2.64) | 1.69 (1.34) |
| coma | 295 | 2.3 (2.05 - 2.58) | 2.3 (213.21) | 2.28 (2.07) | 1.19 (1.02) |
| dyskinesia | 535 | 2.78 (2.55 - 3.03) | 2.78 (599.22) | 2.75 (2.56) | 1.46 (1.33) |

**Abbreviations: PT, preferred term; N, number of adverse event reports; ROR, reporting odds ratio; CI, confidence interval; PRR, proportional reporting ratio; χ², chi-square; EBGM, empirical Bayesian geometric mean; EBGM05, the lower 95% one-sided confidence limit of EBGM; IC, information component; IC025, the lower limit of the 95% credibility interval for IC.**
